# Nitrate-rich beetroot juice offsets salivary acidity following carbohydrate ingestion before and after endurance exercise in healthy male runners

**DOI:** 10.1371/journal.pone.0243755

**Published:** 2020-12-15

**Authors:** Mia C. Burleigh, Nicholas Sculthorpe, Fiona L. Henriquez, Chris Easton

**Affiliations:** 1 Institute of Clinical Health and Exercise Sciences, University of the West of Scotland, Blantyre, Scotland; 2 Institute of Biomedical and Environmental Health Research, University of the West of Scotland, Blantyre, Scotland; National Taipei University of Nursing and Health Sciences, TAIWAN

## Abstract

There have been recent calls for strategies to improve oral health in athletes. High carbohydrate diets, exercise induced dehydration and transient perturbations to immune function combine to increase oral disease risk in this group. We tested whether a single dose of nitrate (NO_3_^-^) would offset the reduction in salivary pH following carbohydrate ingestion before and after an exercise bout designed to cause mild dehydration. Eleven trained male runners ($\dot{V}O_{2\max}$ 53 ± 9 ml∙kg^-1^∙min^-1^, age 30 ± 7 years) completed a randomised placebo-controlled study comprising four experimental trials. Participants ingested the following fluids one hour before each trial: (a) 140 ml of water (negative-control), (b) 140 ml of water (positive-control), (c) 140 ml of NO_3_^-^ rich beetroot juice (~12.4 mmol NO_3_^-^) (NO_3_^-^ trial) or (d) 140 ml NO_3_^-^ depleted beetroot juice (placebo-trial). During the negative-control trial, participants ingested 795 ml of water in three equal aliquots: before, during, and after 90 min of submaximal running. In the other trials they received 795 ml of carbohydrate supplements in the same fashion. Venous blood was collected before and after the exercise bout and saliva was sampled before and repeatedly over the 20 min following carbohydrate or water ingestion. As expected, nitrite (NO_2_^-^) and NO_3_^-^ were higher in plasma and saliva during the NO_3_^-^ trial than all other trials (all *P*<0.001). Compared to the negative-control, salivary-pH was significantly reduced following the ingestion of carbohydrate in the positive-control and placebo trials (both *P* <0.05). Salivary-pH was similar between the negative-control and NO_3_^-^ trials before and after exercise despite ingestion of carbohydrate in the NO_3_^-^ trial (both *P*≥0.221). Ingesting NO_3_^-^ attenuates the expected reduction in salivary-pH following carbohydrate supplements and exercise-induced dehydration. NO_3_^-^ should be considered by athletes as a novel nutritional strategy to reduce the risk of developing acidity related oral health conditions.

## Introduction

Carbohydrate-rich sports products are an important resource for sports performance in certain circumstances and are widely consumed throughout society [1–4]. However, these products contain erosive components such as malic and/or citric acid [5–7] which can be detrimental to oral health due to the rapid drop in salivary pH that is experienced following consumption [5, 7–13]. This acidification of saliva contributes to tooth demineralisation and the cariogenic potential of carbohydrate ingestion [6, 13–16]. Professional athletes have an increased risk for developing oral diseases, likely due to frequent consumption of carbohydrate drinks and gels to maintain adequate energy levels [17–20]. Furthermore, the capacity of saliva to normalise oral pH and resist oral disease development may be limited in this population due to exercise-associated bouts of dehydration, transient perturbations to immune function and reduced salivary flow-rates induced by mouth breathing during exercise [17–19, 21–23]. Despite recent calls to address poor oral health in athletes [20], the problem remains widespread and additional preventative measures are sought after [17].

One solution could be to increase the intake of foods and beverages that elevate salivary pH [24–26]. Our group and others have shown that increasing the nitrate (NO_3_^-^) content of the diet for several days with NO_3_^-^ rich beetroot juice can increase salivary pH [27, 28]. This is significant because a neutral pH is considered optimal for a healthy oral environment and the risk of enamel erosion rises if pH falls below 5.5 [29]. Furthermore, individuals with naturally high salivary NO_3_^-^ and NO_2_^-^ levels are reported to have lower incidences of caries [30, 31]. When ingested, NO_3_^-^ is absorbed in the upper gastrointestinal tract and a portion re-enters the mouth following excretion from the salivary glands. Here, bacteria with NO_3_^-^ reductase genes can perform a stepwise reduction of NO_3_^-^ to nitrite (NO_2_^-^) and nitric oxide (NO) [32]. The increase in salivary pH that follows dietary NO_3_^-^ supplementation is suggested to be a consequence of the bactericidal effects of NO_2_^-^ and NO on saccharolytic bacteria residing in the oral cavity and/or the production of ammonium [33–35]. Indeed, *in vitro* research suggests that elevated levels of NO_2_^-^ and NO_3_^-^ can substantially reduce salivary acidity in response to glucose [33, 36] and that the genera *Rothia* may be a probiotic in the presence of NO_3_^-^ via consumption of lactate [37]. However, it is currently unknown whether this beneficial effect would be present *in vivo*.

NO also has positive effects on the regulation of mucosal blood flow and mucus generation [38], regulation of smooth muscle contraction [39], cerebral blood flow [40], glucose homeostasis [41], and mitochondrial function [42] and is known to be beneficial for cardiovascular health [43, 44]. Furthermore, NO_3_^-^ rich beetroot juice supplementation is often reported to have positive effects on aerobic exercise performance [45, 46], potentially due to a reduction in oxygen utilisation which improves the efficiency of muscle contraction [47–49]. The ergogenic effects of NO_3_^-^ are also apparent in sprint performance and power output which may be a consequence of enhanced muscle contractile function from improved Ca^2+^ handling and/or enhanced blood flow to type II muscle fibres [48, 50, 51]. Collectively, these studies support the notion that there may be myriad benefits for athletes following NO_3_^-^ supplementation.

Whilst convincing evidence exists to suggest that several days of NO_3_^-^ supplementation can increase salivary pH [27, 28], no previous study has examined *in vivo* whether prior ingestion of NO_3_^-^ can offset the decline in salivary pH following carbohydrate supplementation [10, 13, 14] and whether these effects are influenced by prior exercise. Furthermore, it is unclear how soon the increase in pH occurs after ingestion of NO_3_^-^ as previous work has focussed only on chronic supplementation rather than acute effects. This dosing information is critical for athletes wishing to incorporate the oral health benefits of NO_3_^-^ as part of their nutritional strategy. Therefore, the primary aim of this study was to determine the effects of a single dose of NO_3_^-^ rich beetroot juice on salivary pH following carbohydrate supplementation at rest and after endurance exercise. The results confirm that ingesting NO_3_^-^ increases salivary-pH and extend these findings by showing that this occurs in an acute timeframe. In addition, salivary pH remained elevated following carbohydrate ingestion and exercise, suggesting that NO_3_^-^ supplementation attenuates the reduction in salivary pH associated with carbohydrate supplements and exercise-induced dehydration.

## Methods

### Participants and ethical approval

Participants were recruited via advertisement of the study on social media outlets and by contacting running club secretaries. Females were not recruited for this study due to evidence suggesting that hormonal fluctuations affect salivary composition and pH [52, 53]. Eleven trained male runners ($\dot{V}O_{2\max}$ 53 ± 9 ml∙kg^-1^∙min^-1^, age 30 ± 7 years, stature 179 ± 7 cm, and body mass 86.9 ± 14.1 kg) volunteered to take part in the study and provided written informed consent. The estimated sample size (n = 10) was based on the expected difference in the primary outcome (salivary pH) measurement using data previously collected in our laboratory following the ingestion of NO_3_^-^ (Measurement 1: 7.13 ± 0.54 AU Measurement 2: 7.39 ± 0.68 AU; R = 0.95) with an α set at 0.05 and power 0.8 [28]. Eleven participants were recruited to account for potential dropout.

All participants were in good cardiovascular and oral health and did not report any use of antibacterial mouthwash or antibiotics for at least 6 months prior to study commencement. They were free from non-prescription medication including those known to interfere with stomach acid production and were not taking any prescribed medication. Health status was confirmed by completion of a medical questionnaire and The World Health Organisation’s oral health questionnaire was used to ascertain oral health status.

The study was approved by the School of Health and Life Sciences Ethics Committee at The University of the West of Scotland. All procedures described were conducted in accordance with the Declaration of Helsinki 1974 and its later amendments.

### Experimental design

A randomised, placebo-controlled study design was used to determine the effects of NO_3_^-^ rich beetroot juice on salivary pH following the ingestion of carbohydrate-based sports supplements. Participants visited the laboratory a total of five times with a minimum of 7 days between visits. During the first visit, participants completed an incremental treadmill running test for measurement of maximal oxygen utilisation ($\dot{V}O_{2\max}$) and the gas-exchange threshold (GET). Participants were also familiarised with the protocols for the four subsequent experimental trials.

**Fig 1:**
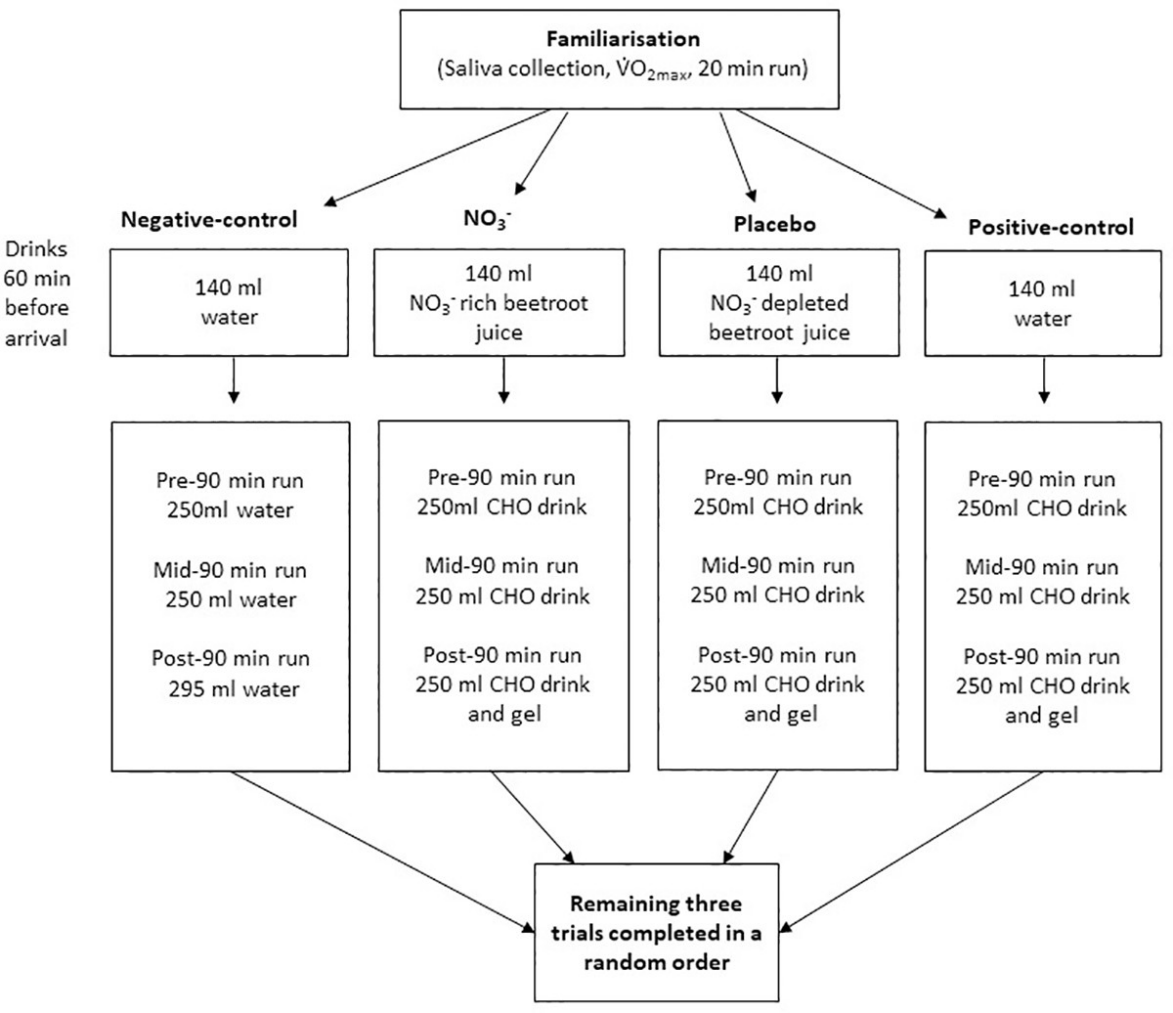
Study flow chart showing details of drinks consumed before and during each experimental trial.

As detailed in Fig 1, participants ingested various combinations of fluids, before, during, and after exercise. One hour prior to the trial, participants ingested either: (a) 140 ml of water (negative-control), (b) 140 ml of water (positive-control), (c) 140 ml of NO_3_^-^ rich beetroot juice (~12.4 mmol NO_3_^-^) (NO_3_^-^ trial) or (d) 140 ml NO_3_^-^ depleted beetroot juice (placebo-trial). During the negative-control trial, participants ingested 795 ml of water in three equal aliquots: before, during, and after 90 min of submaximal running. The order of the experimental trials was determined using a computer programme [54]. The NO_3_^-^ rich and NO_3_^-^ depleted versions of the beetroot juice were identical in taste, appearance, and packaging to ensure both participants and investigators were blind to the order of these trials. It was not possible to blind participants to the negative and positive-control trials.

### Procedures

#### Familiarisation trial

Participants were initially familiarised with the procedures for collecting stimulated and unstimulated samples of saliva to ensure a standardised collection technique in subsequent trials. Following this, participants completed a graded exercise test to exhaustion on a motorised treadmill (Woodway, pps 55sporti, Waukesha, Wisconsin, USA,). Heart rate (HR) was recorded continuously by telemetry (Polar Electro, Oy, Finland). Expired gas was measured via indirect calorimetry (Metamax 3B, Cortex, Leipzig/Germany) and analysed for respiratory variables to enable the calculation of $\dot{V}O_{2\max}$ and GET using the V-slope method [55]. Following a 20 min rest period, participants then undertook 20 min of treadmill running at a velocity equivalent to 90% of the GET.

#### Experimental trials

**Fig 2:**
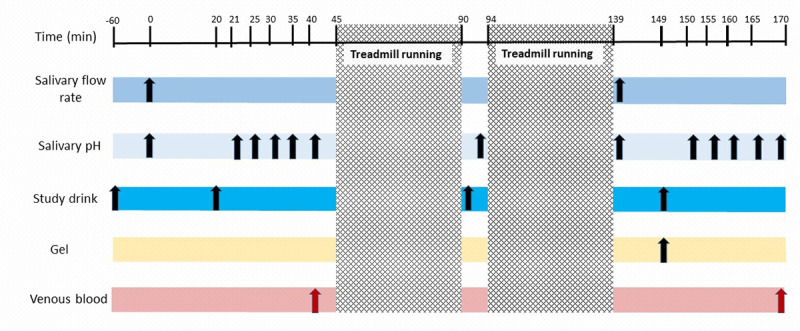
The schematic details sample collection timings and submaximal running in the experimental trials. Trials were identical with the exception of the drinks consumed (See Fig 1).

A schematic overview of the experimental trials is provided in Fig 2. Two hours prior to the trial, participants ate a self-selected light meal and drank 500 ml of water. No other food or fluid was allowed thereafter, excluding the drink(s) which had been allocated for consumption (Fig 1). The drinks used were: NO_3_^-^ rich beetroot juice (~12.4 mmol NO_3_^-^, James White Drinks Ltd., Suffolk, England, total 30.8 g carbohydrate (30.8 g sugar), pH 4.0), NO_3_^-^ depleted beetroot juice (Placebo shots, James White Drinks Ltd., Suffolk, England, total 30.8 g carbohydrate (30.8 g sugar), pH 4.0), and water (Nestle Pure Life, Nestle, Vevey, Switzerland, pH 8). All food and drink consumed in the three days prior to the first experimental trial was recorded and repeated before each subsequent visit.

Upon arrival, nude body mass was recorded, and hydration status assessed via urine osmolality (Osmocheck, Vitech, Scientific Ltd. West Sussex, UK). Following this, stimulated and unstimulated salivary flow-rate were measured over 5 min as described elsewhere [56]. Participants then ingested 250 ml of water in the negative-control trial or 250 ml of carbohydrate drink (Powerade Berry and Tropical flavoured drink, The Coca-Cola Company, Atlanta, USA total 30 g carbohydrate, (30 g sugar), pH 3.2). Further samples of unstimulated saliva were collected immediately before and repeatedly for 20 min after the fluid ingestion. On completion of saliva sampling, 4 ml of venous blood was collected via venepuncture from the forearm in vacutainer tubes containing ethylenediaminetetraacetic acid (BD vacutainer K2E 7.2mg, Plymouth, U.K.). Samples of whole blood were immediately centrifuged for 10 min at 4000 rpm at 4°C (Harrier 18/80, Henderson Biomedical. UK) following collection.

Participants then completed 90 min of treadmill running at a velocity equivalent to 90% of the GET. Heart rate and rating of perceived exertion using the Borg scale were recorded every 5 min. The exercise was continuous with the exception of a 4 min break midway to allow further drink consumption (Fig 1). Following completion of the exercise bout, participants ingested a further 250 ml of water (negative-control trial only) or another 250 ml aliquot of carbohydrate drink and a carbohydrate gel (Torq Banana and Orange Energy gel, Torq LTD. Trewern, UK, total 28.8 g of carbohydrate, (9.6 g sugar), pH 2.8) in all other trials. Samples of unstimulated saliva and venous blood were then collected as previously described. Nude body mass was re-measured and sweat-rate calculated after correcting for fluid intake and urinary output. All samples of saliva and plasma were stored at -80 ˚C and analysed for [NO_3_^-^], [NO_2_^-^], and pH (saliva only).

#### Analysis of saliva and plasma samples

The pH of saliva samples was measured in duplicate with a circular electrode pH-meter 1140 Mettler Toledo (Greisensee, Switzerland) which has a precision of 0.01 pH unit. The measured pH value was not accepted until an unchanged pH value was observed for a period of at least 7 s. Calibration of the pH meter was performed before analysis and after every 10 samples using buffers with known pH (4.01 and 7.00). The electrode was rinsed with 18 Ω deionised water between samples.

For the analysis of plasma and saliva [NO_2_^-^], tri-iodide reagent (2.5 ml glacial acetic acid, 0.5 ml of 18 Ω deionised water, and 25 mg sodium iodide) was placed in a glass purge vessel heated to 50°C and connected to a NO analyser (Sievers NOA 280i, Analytix, UK). A standard curve was created by injecting 100 μL of NO_2_^-^ solutions at various concentrations up to 1000 nM (plasma) and 3000 nM (saliva). Samples were thawed in a water bath at 37°C and 100μL of the sample was injected immediately into the purge vessel in duplicate. Saliva samples were initially diluted with deionised water at a ratio of 1:100 before injection. The NO_2_^-^ content was calculated via the area under the curve using Origin software (version 7.1).

For the analysis of [NO_3_^-^], vanadium reagent (24 mg of vanadium tri-chloride and 3 ml of 1 M hydrochloric acid) was placed into the purge vessel and heated to 90°C. A standard curve was created by injecting 10–25 μL NO_3_^-^ solutions at concentrations up to 100 μM for both plasma and saliva. Plasma samples were initially de-proteinised using 1 M zinc sulfate (ZnSO_4_) at 1:10 w/v and 1 M sodium hydroxide (NaOH) at a 1:1 ratio. 200 μL of plasma was added to 400 μL of ZnSO_4_ and 400 μL of NaOH. Each sample was vortexed for 30 s prior to being centrifuged for 5 min at 4000 rpm and the supernatant was injected into the purge vessel. The NO_3_^-^ concentration was calculated as previously described for NO_2_^-^.

#### Calculations

To assess whether beetroot juice influenced salivary pH overall following carbohydrate ingestion the area under the curve (AUC) was calculated in all four trials using the trapezoidal method. The incremental AUC was also calculated to determine whether beetroot juice influenced the magnitude of the delta change in pH from baseline (0 min) in response to carbohydrate. These analyses were conducted at both the pre-exercise and post-exercise time points in all experimental trials.

#### Statistics

Jamovi (version 1.0.0, 2019, www.jamovi.org) was used for statistical analysis. GraphPad Prism version 5 (GraphPad Software Inc., San Diego, USA) was used to create the figures. The distributions of data were assessed using the Shapiro Wilk test; non-parametric tests were used where data were not normally distributed. A one-factor repeated-measures ANOVA was used to assess the main effect of condition (NO_3_^-^ trial, negative-control trial, positive-control trial, and placebo-trial) on, mean HR, mean rating of perceived exertion, pre-exercise urine osmolality, and sweat-rate. A two-factor repeated-measures ANOVA was used to assess the effects of exercise (pre and post-exercise), condition, and the interaction effects on NO_3_^-^ and NO_2_^-^ levels in plasma and saliva, salivary pH AUC, and salivary flow-rate. *Post-hoc* analysis was conducted following a significant main effect or interaction using paired samples t-tests with Tukey’s correction for multiple pairwise comparisons. The alpha level for declaring statistical significance was set at *P*≤0.05. Data are presented as mean ± standard deviation (SD) unless otherwise stated. Probability values are expressed with 95% confidence intervals (95%CI) where appropriate.

## Results

### NO_3_^-^ and NO_2_^-^ levels

**Fig 3:**
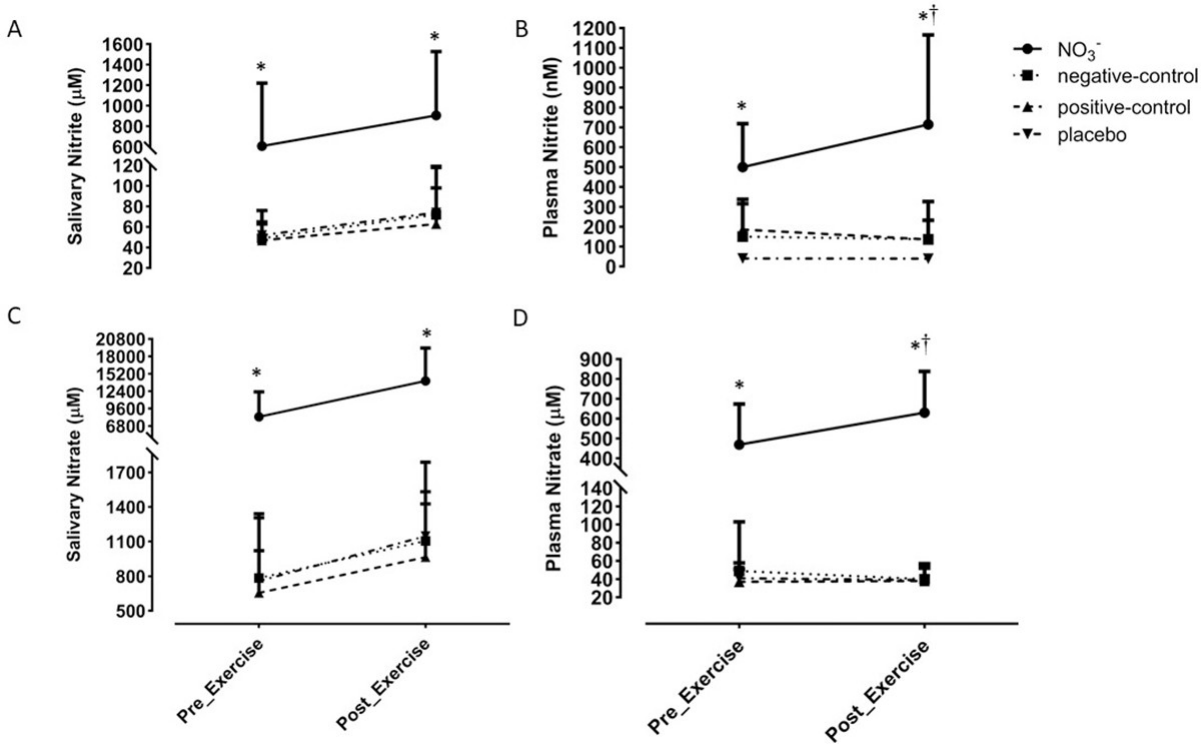
Salivary (NO_2_^-^) **(A)**, plasma (NO_2_^-^) **(B)**, salivary (NO_3_^-^) **(C)**, and plasma (NO_3_^-^) **(D)** measured pre- and post-exercise in each trial. * denotes a significant difference from all other trials and † denotes a significant increase from the within trial pre-exercise measurement. For clarity, only the upper SD bars are displayed.

Saliva and plasma NO_3_^-^ and NO_2_^-^ data are presented in Fig 3. There was a significant main effect of condition on salivary NO metabolites (both *P*<0.001). There were no effects of time or condition by time interactions for salivary NO metabolites (all *P*>0.05). There was a significant main effect of condition (*P*<0.001), time (*P*<0.02) and a condition by time interaction (*P*<0.001) for plasma [NO_2_^-^] and [NO_3_^-^]. *Post-hoc* analysis showed that [NO_2_^-^] and [NO_3_^-^] were higher at both the pre and post-exercise time-points in the NO_3_^-^ trial than in all other trials (all *P*<0.001). There were no differences in the levels of these NO metabolites between the negative-control, positive-control and placebo trials (all *P*>0.05). There was a significant increase in plasma [NO_2_^-^] (*P* = 0.01, 95%CI 372–629 nM) and plasma [NO_3_^-^] (*P*<0.001, 95%CI 406–533 μM) from pre- to post exercise in the NO_3_^-^ trial but not the other trials (all *P*>0.05). Salivary NO metabolites did not change from pre- to post-exercise in any trial (all *P*>0.05).

### Salivary pH

**Fig 4:**
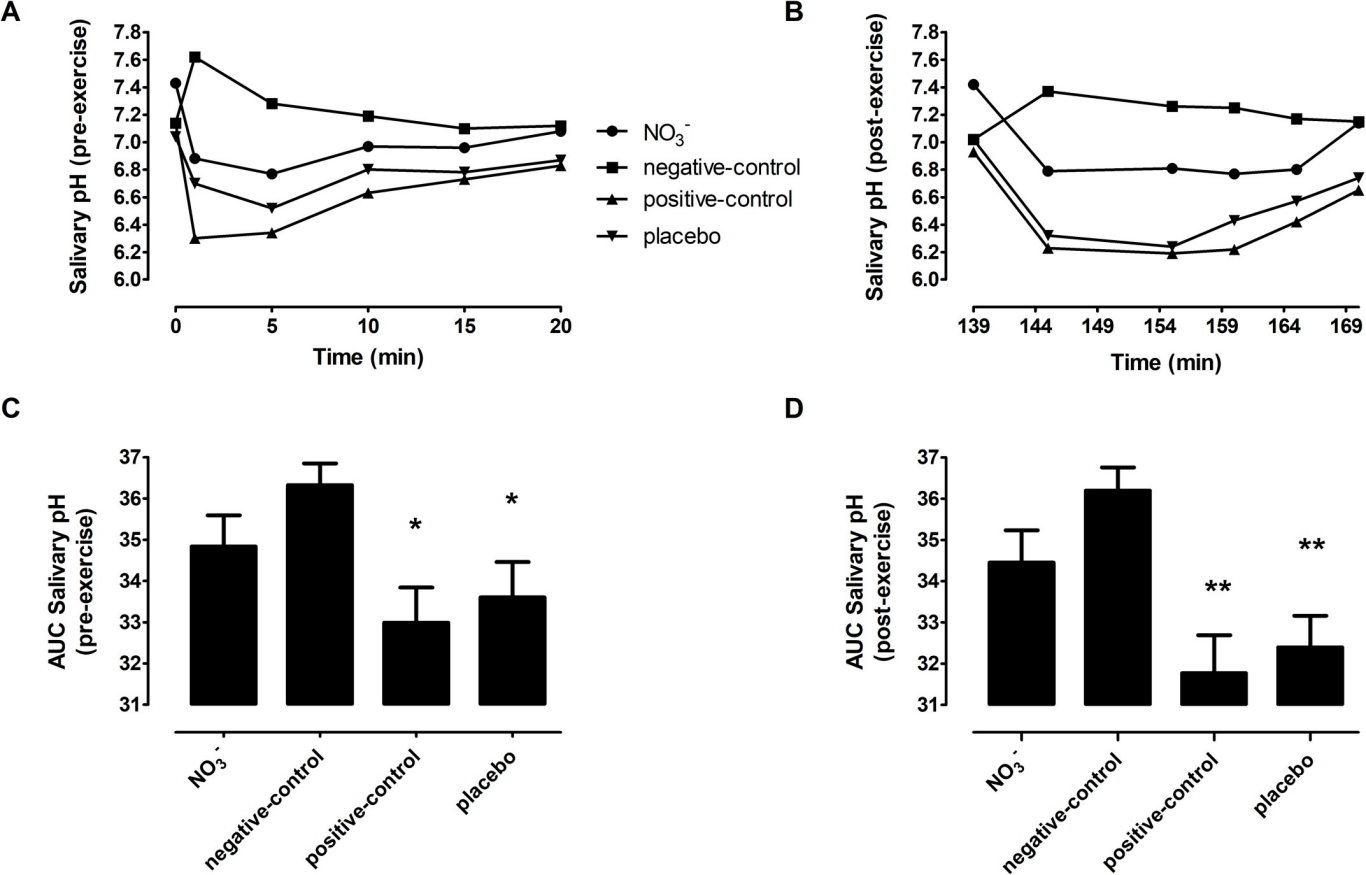
**The line graphs display the salivary pH response to carbohydrate or water in each trial over the 20 min post ingestion period both pre- and post-exercise. (A)** salivary pH pre-exercise, **(B)** salivary pH post-exercise. The bar charts display the AUC of these responses: **(C)** Pre-exercise AUC, **(D)** Post-exercise AUC. * indicates that the AUC of pre-exercise salivary pH was smaller in the placebo and positive-control trials than in the negative-control-trial. ** shows that the salivary pH AUC in both the NO_3_^-^ and negative-control trials was greater than the positive-control and placebo trials. Error bars have been removed from panels A and B for clarity.

Salivary pH data are presented in Fig 4. There was a significant main effect of condition (*P* = 0.01) on salivary pH. *Post-hoc* analysis revealed that salivary pH at baseline was higher in the NO_3_^-^ trial than in all the other trials (all *P*<0.02, NO_3_^-^ trial 7.43 ± 0.37, negative-control trial 7.14 ± 0.26, positive-control trial 7.14 ± 0.26, placebo trial 7.04 ± 0.31, Fig 4A). Immediately after exercise, salivary pH remained higher in the NO_3_^-^ trial than the other trials (all *P*<0.02, NO_3_^-^ trial 7.42 ± 0.41, negative-control trial 7.02 ± 0.19, positive-control trial 6.93 ± 0.22, placebo trial 7.02 ± 0.21, Fig 4B). There were no other differences in salivary pH between trials at these time points (all *P*>0.05).

In the negative-control trial, the ingestion of water tended to increase salivary pH before (Fig 4A) and after exercise (Fig 4B). In the other trials, the ingestion of carbohydrate tended to decrease salivary pH at the same time-points. The overall response in salivary pH was assessed using the trapezoidal AUC. These analyses demonstrated there was a significant main effect of condition (*P* = 0.002) and time (*P*<0.001) on the trapezoidal AUC for salivary pH but no interaction effect (*P* = 0.079). The trapezoidal AUC was greater pre- and post-exercise in the negative-control trial and the NO_3_^-^ trial compared to the other two trials (all *P*<0.03, Fig 4C and 4D). In the placebo condition, the trapezoidal AUC for salivary pH was lower at the post-exercise time point compared to pre-exercise (*P* = 0.007) but was not different in the other conditions. Further calculation of the incremental AUC enabled us to ascertain whether ingestion of beetroot juice influenced the magnitude of the change in salivary pH from baseline after the of carbohydrate load. There was a main effect of condition (*P* = 0.024), time (*P* = 0.024), and condition time interaction (*P* = 0.003) on the incremental AUC for salivary pH. The incremental AUC was also greater pre- and post-exercise in the negative-control and the NO_3_^-^ trial compared to the two other trials (all *P*<0.035). In the positive-control and placebo conditions, the incremental AUC values were both lower at the post-exercise time point compared to pre-exercise (positive-control *P* = 0.043 and placebo *P* = 0.013).

### Salivary flow-rate

Data for stimulated and unstimulated salivary flow-rate values are presented in Table 1. The unstimulated and stimulated salivary flow-rates did not differ between conditions (all *P*>0.05). The unstimulated salivary flow-rate did not change from pre- to post-exercise in any trial (all *P*>0.05). There was a significant main effect of time on the stimulated salivary flow rate (*P*<0.001). Stimulated salivary flow-rate was reduced from pre- to post-exercise in both the positive-control (*P* = 0.02, 95%CI 0.79–1.43 ml/min) and the placebo trial (*P*<0.001, 95%CI 0.90–1.5 ml/min) but did not change in the negative-control (*P* = 0.177) or the NO_3_^-^ trial (*P* = 0.086).

**Table 1:** Unstimulated and stimulated salivary flow-rate values in each trial are displayed as mean *±* SD.

Trial	Unstimulated Salivary Flow-rate (ml/min)		Stimulated Salivary Flow-rate (ml/min)	
	Pre-exercise	Post-exercise	Pre-exercise	Post-exercise
NO_3_^-^	0.52 ± 0.23	0.60 ± 0.62	1.37 ± 0.65	0.99 ± 0.45
Negative-control	0.46 ± 0.29	0.41 ± 0.26	1.26 ± 0.50	0.93 ± 0.48
Positive-control	0.43 ± 0.19	0.32 ± 0.19	1.35 ± 0.60	0.88 ± 0.43 *
Placebo-trial	0.53 ± 0.20	0.33 ± 0.19	1.50 ± 0.63	0.95 ± 0.45 *

* Shows that stimulated salivary flow-rate was significantly lower following exercise in the positive-control and placebo trials.

### Effects of exercise on HR, RPE, body mass, and urine osmolality

There was no difference in either mean HR (156 ± 19 bpm) or RPE (12 ± 2) between trials (all *P*>0.05). On average, participants had a sweat rate of 1.2 ± 0.2 L/h which, following correction for fluid intake and urine loss, resulted in a 3 ± 1% reduction in body mass. Urine osmolality increased from 366 ± 190 mOsm/kg before exercise to 595 ± 164 mOsm/kg after exercise, (*P*<0.001). The changes in body mass and urine osmolality did not differ between trials (all *P*>0.05).

## Discussion

We, and others, have previously reported that supplementing the diet with NO_3_^-^ over a number of days can increase the pH of saliva. Here, we explored whether a single dose of NO_3_^-^ rich beetroot juice could offset the rapid decline in salivary pH that typically follows the ingestion of carbohydrate supplements in athletes [5, 8–10, 57, 58]. As expected, when a cohort of trained runners consumed carbohydrate sport drinks before, during, and after exercise, they experienced a significant reduction in salivary pH. The principal finding of this study was that prior ingestion of a single bolus of NO_3_^-^ rich beetroot juice was sufficient to offset this decline in salivary pH. These effects appear to be mediated specifically by NO_3_^-^ as NO_3_^-^ depleted beetroot juice did not influence the salivary pH response to carbohydrate ingestion. Therefore, NO_3_^-^ rich beetroot juice may offer some promise as a potential solution to protect oral health in athletes.

### Plasma and saliva nitric oxide metabolites

In line with our previous research [45, 59], the ingestion of NO_3_^-^ rich beetroot juice increased the concentration of NO_3_^-^ and NO_2_^-^ in the saliva and plasma within 1 hour. The levels of these NO metabolites remained significantly elevated in saliva and increased in the plasma following 90 min of treadmill running. The increase in plasma [NO_2_^-^] and [NO_3_^-^] from pre-exercise (100 minutes after ingestion of NO_3_^-^ rich beetroot juice) to post-exercise (219 minutes after ingestion of NO_3_^-^ rich beetroot juice) reflects the established pharmacokinetics of these metabolites [59, 60] While plasma NO_3_^-^ typically peaks 1–2 h after ingestion of beetroot juice, the rise and peak in plasma NO_2_^-^ occurs more slowly (2–3.5 h) [45, 60, 61] as commensal microbes of the oral microflora progressively reduce the NO_3_^-^ in saliva to NO_2_^-^. It is also possible that exercise may have impacted directly on plasma [NO_2_^-^] due to postural-induced shifts in plasma volume [62], dehydration [63], and/or increased conversion of NO_2_^-^ to NO [64]. However, neither [NO_2_^-^] or [NO_3_^-^] were altered from pre- to post-exercise in the placebo or control trials suggesting the direct impact of exercise on plasma NO metabolites is small.

### Salivary flow-rate

In line with previous research [5, 8, 9], the ingestion of a carbohydrate-rich sports drink resulted in a significant and rapid (within 1 min) reduction in salivary pH at rest. This is a consequence of citric acid which reduces the pH of the drink to 3.2 and immediately acidifies the saliva during drinking [5, 6]. Citric acid-based drinks have been shown to be particularly damaging to tooth enamel [65] although the saturation of minerals and the adhesiveness of the solution will also influence the erosive potential [66]. Exercise-induced reductions in saliva production may increase the risk for dental caries following carbohydrate ingestion as saliva provides the initial buffer against erosion by helping to clear acids from the mouth [6, 58]. In the present study, stimulated salivary flow-rate was significantly reduced (<1 ml/min) in the positive-control and placebo conditions following exercise. This may be due to a reduced availability of saliva following increased ventilation for a prolonged period and/or substantial exercise-induced dehydration amounting to ~3% of body mass. This is important in the context of frequent exercise training as salivary flow-rates of ≤1 ml/min have been associated with a fivefold increase in dental erosion rates [67]. On the other hand, there was no significant reduction in the stimulated saliva flow rate in the NO_3_^-^ trial suggesting NO_3_^-^ rich beetroot juice may help preserve saliva production. While the mechanism for this is unclear, NO_3_^-^ supplementation has been shown to induce vasodilation and increase blood flow during exercise [51]. It must be acknowledged, however, that stimulated saliva production was also not reduced significantly after exercise in the negative-control trial (water only) and the unstimulated salivary flow-rate did not change from pre- to post-exercise in any condition. This should be the subject of a more focussed investigation as to ascertain whether dietary NO_3_^-^ can influence salivary flow-rate via NO mediated vasodilation at the vascular beds of the salivary glands [68].

### Salivary pH

A notable finding in the present study was that NO_3_^-^ rich beetroot juice increased baseline salivary pH which substantially offset the salivary acidity that followed the ingestion of carbohydrate supplements in the hours following ingestion. Interrogation of the incremental AUC data demonstrates that the preservation of saliva pH was not solely a consequence of the elevated pH at baseline. Rather, NO_3_^-^ rich beetroot juice also reduces the rate by which salivary pH declines after the ingestion of a carbohydrate load. This is particularly remarkable when one considers that the beetroot juice itself is carbohydrate-rich and has a pH of 4.0. These effects must be mitigated by NO_3_^-^ given that NO_3_^-^ depleted beetroot juice (placebo trial) had no measurable impact on salivary pH. This supports the findings of previous *in-vitro* work which showed that NO_3_^-^ elevated the pH of saliva samples in the presence of glucose [33]. It also extends our previous research (20) and the work of others [69, 70] that reported longer term (1 week or greater) supplementation with dietary NO_3_^-^ increases salivary pH humans for which there are several There are several plausible explanations as to how chronic supplementation NO_3_^-^ could increase salivary pH. For example, it is clear that increasing the concentration of NO_2_^-^ in saliva (following reduction from NO_3_^-^) for several days can have a profound antibacterial effect [71]. When NO_2_^-^ encounters the acidic environment of the mouth, it can be subject to a stepwise reduction to NO. The NO can pass through the bacterial cell membrane and damage the microorganism through inhibition of DNA synthases and other effects [71]. Increasing the concentration of NO metabolites in the oral cavity can also repress bacterial acid fermentation and/or increase alkali production [30, 34, 72]. What is less clear is how these effects were mitigated within the acute time frame (1 h after ingestion) observed in this study.

In support of our findings a recent *in vitro* study by Rosier and colleagues [36] exposed saliva from healthy donors to NO_3_^-^ and glucose. These authors found that after just 5 h nitrate had increased ammonium, pH, and the health associated genera *Neisseria*. Simultaneously lactate and several bacteria associated with caries, halitosis, and periodontitis were reduced. Our present data confirms for the first time that, *in vivo*, NO_3_^-^ offsets the acidification of saliva following carbohydrate supplements in the hours following ingestion, presumably through similar mechanisms to those reported by Rosier et al. [36]. This finding is important because limiting acidification of saliva will reduce the amount of time teeth are exposed to an acidic environment and potentially reduce the development of dental disease [36].

From a practical perspective, our study informs dosing strategies by showing that long periods of supplementation are not needed to achieve the potential protective effects of NO_3_^-^ on oral health and that acidity is suppressed for at least 3 h 50 min following NO_3_^-^ ingestion. This information is particularly relevant to the athletic community as athlete health is at the forefront of all structures and processes which feed into performance outcomes [73] and improving oral health in this group presents a unique challenge, because carbohydrate supplementation and training practices are not easily modifiable without incurring a performance decrement [2, 22, 74, 75].

### Limitations

It is important to acknowledge that, although we present some important findings, there are some limitations. Firstly, our small sample of healthy trained runners were homogenous, and it is not clear whether these findings would translate to females or other populations. Secondly, the exercise protocol and the carbohydrate ingestion regimen employed in this study may not reflect those of all athletes during training or competition. Lastly our study does not reveal a definitive mechanism for the acute increase in pH after NO_3_^-^ supplementation although the recent *in vitro* work of Rosier and colleagues [36] both supports our findings and provides potential mechanistic evidence for the results. It would be of interest to repeat our study with measurements of arginine deaminase and urease [36], plus metagenomic and metatranscriptomic analysis to assess the abundance and activity of bacteria in response to combined NO_3_^-^ and CHO supplementation in the hours following administration.

### Implications and future directions

In response to the recent calls to address poor oral health in athletes [20, 74], the present study demonstrates that NO_3_^-^ supplementation is a practical and effective method of increasing salivary pH. Using a protocol that was designed to mimic a typical training session of an endurance athlete, NO_3_^-^ rich beetroot juice suppressed the acidification of saliva that arose from the ingestion of sports drinks and carbohydrate gels. These findings have practical application for athletes who are interested in protecting oral health, especially when one considers the potential ergogenic effects of beetroot juice [47, 76, 77]. However, athletes should always balance the financial costs and possibility of gastrointestinal issues before adopting any new supplementation strategy. A diet first approach may be more appropriate than supplementation and this should be further investigated. Importantly, we did not observe gastrointestinal issues in participants who had not previously experienced these symptoms during training and/or competition, but this should always be considered at an individual level.

Future work should employ a longitudinal design to explore how chronic dietary NO_3_^-^ supplementation influences the oral health of athletes. There should also be additional focus on female athletes as salivary pH is known to fluctuate with the menstrual cycle and female oral health is known to deteriorate post-menopause [78]. This means that females, particularly those who are older, may be at higher risk of acid erosion from carbohydrate supplementation [53].

## Conclusions

A single dose of NO_3_^-^ rich beetroot juice increased salivary pH for several hours following ingestion. This suppressed the acidification of saliva that followed ingestion of carbohydrate-rich supplements before and after a sustained period of exercise which resulted in mild dehydration. For athletes who regularly consume carbohydrate and are exposed to high training loads, ingestion of NO_3_^-^ may provide a practical and effective method to protect their oral health whilst maintaining training volume and intensity.

## Supporting information

S1 Dataset (XLSX)
